# Efficient Matching-Based Parallel Task Offloading in IoT Networks

**DOI:** 10.3390/s22186906

**Published:** 2022-09-13

**Authors:** Usman Mahmood Malik, Muhammad Awais Javed, Jaroslav Frnda, Jan Rozhon, Wali Ullah Khan

**Affiliations:** 1 Department of Electrical and Computer Engineering, COMSATS University Islamabad, Islamabad 45550, Pakistan; 2 Department of Electrical Engineering, National University of Science and Technology (NUST), Islamabad 44000, Pakistan; 3 Department of Quantitative Methods and Economic Informatics, Faculty of Operation and Economics of Transport and Communications, University of Zilina, 01026 Zilina, Slovakia; 4 Department of Telecommunications, Faculty of Electrical Engineering and Computer Science, VSB Technical University of Ostrava, 70800 Ostrava, Czech Republic; 5 Interdisciplinary Centre for Security, Reliability and Trust (SnT), University of Luxembourg, 1855 Luxembourg, Luxembourg

**Keywords:** Internet of Things, fog computing, task offloading, partial task offloading, matching theory, externalities problem

## Abstract

Fog computing is one of the major components of future 6G networks. It can provide fast computing of different application-related tasks and improve system reliability due to better decision-making. Parallel offloading, in which a task is split into several sub-tasks and transmitted to different fog nodes for parallel computation, is a promising concept in task offloading. Parallel offloading suffers from challenges such as sub-task splitting and mapping of sub-tasks to the fog nodes. In this paper, we propose a novel many-to-one matching-based algorithm for the allocation of sub-tasks to fog nodes. We develop preference profiles for IoT nodes and fog nodes to reduce the task computation delay. We also propose a technique to address the externalities problem in the matching algorithm that is caused by the dynamic preference profiles. Furthermore, a detailed evaluation of the proposed technique is presented to show the benefits of each feature of the algorithm. Simulation results show that the proposed matching-based offloading technique outperforms other available techniques from the literature and improves task latency by 52% at high task loads.

## 1. Introduction

The Internet of Things (IoT) is the major source of many upcoming applications related to intelligent transportation, health care, and smart grids [1,2,3,4]. IoT relies on many new technologies such as 6G communications, fog computing, and data analytics to provide ubiquitous connectivity, fast computing services, and intelligent application-related decision-making [5,6,7,8].

Fog computing holds key importance in future IoT applications as it helps with the fast computing of different application tasks. Fog computing offers many benefits compared to traditional cloud computing [9,10,11]. Due to being closer to the edge and distributed in nature, fog nodes provide quicker and more reliable computation of tasks. However, efficient task offloading from IoT nodes to fog nodes is a major challenge that needs to be addressed.

Partial Offloading to Multiple Helper (POMH) is a task offloading technique that offers parallel computation of tasks and improves task latency. The tasks are divided into sub-tasks and passed to different fog nodes in parallel so that the overall computation time is reduced [12]. The focus of this paper is to utilize graph matching theory to address the resource allocation challenges in POMH. The matching theory has the advantage of allocating resources in a stable manner while considering the preferences of agents [13]. However, POMH is complex and involves decisions that require coordination among multiple stakeholders, such as task splitting and task offloading.

In POMH, the resource allocation problem is exacerbated because the sub-tasks of a task are processed at different computing devices, each of which takes a different amount of time to complete. The sub-task that takes the longest to complete determines the task completion time [14]. For efficient utilization of assigned computation resources, all sub-tasks of a task must be completed at the same time. To do this, the size of sub-tasks needs to be adjusted proportionately to the computation resources allocated to that task.

This indicates that when we use the matching theory for resource allocation in POMH, the size of sub-tasks will vary with every allocation and cancellation of the potential match. This change in sub-task size changes the time and energy estimates for that task, generating variations in preference order of the tasks for the helping devices. In matching theory, this is an example of externalities, where players of one or both sets have dynamic preference profiles, and the decision of one player influences the decision of all other players [15].

Solving the externalities problem in matching theory to make stable matching decisions is a difficult proposition that has long been a research focus, with few solutions proposed. Externalities problems are cyclic in nature and difficult to contain [16]. Jinpeng Ma (JM) [17] proposed that if we first find stable matching assignments while ignoring the externalities problem and then update the matched pairs to solve blocking pairs that arise due to changes in player preference profiles, the externalities problem can be solved in polynomial time with a convergence probability of one. In this paper, we used the JM concept to solve the externalities problem in POMH task offloading using the many-to-one matching technique. We use the Deferred Acceptance Algorithm (DAA) [18] to obtain initial stable matching assignments while ignoring the externalities problem. This stable matching assignment is then updated to solve the externalities problem using the novel Stable Matching Update Algorithm (SMUA). The main contributions of this paper can be summarized as:

1. To make the best use of the limited computation resources available on helper devices, we use POMH and formulate the resource allocation problem as a many-to-one matching problem. A task is divided into multiple sub-tasks that are processed concurrently at the task-originating device and multiple helper devices to reduce task completion time.
2. To solve the externalities problem and produce stable matching assignments for POMH task offloading, we develop a novel many-to-one Stable Matching Update Algorithm (SMUA) based on the JM algorithm [17]. To the best of our knowledge, this is the first work that addresses the externalities problem in matching theory for resource allocation in POMH.

The remainder of the paper is as follows. In Section 2, we discuss related work on POMH and the externalities problem in matching theory. We provide a system model and formulate the problem in Section 3. In Section 4, we propose a solution based on the matching technique. We compare the performance of our proposed technique in Section 5, and we conclude in Section 6.

## 2. Related Work

In this section, we discuss a literature review of fog computing to understand and summarize work done by researchers in areas of (a) partial offloading of a task to a single helper device, (b) partial offloading of a task to multiple helper devices, and (c) the externalities problem of matching theory and how scholars have recommended solving this problem. Previous work on partial task offloading in fog computing and contributions of this paper are summarized in Table 1.

### 2.1. Partial Offloading to Single Helper

Studies on task offloading with a single helper have focused on both binary and non-binary decision-making. In binary decision-making, the entire task is either offloaded to another device or retained for local computation, whereas in non-binary decision-making, i.e., partial offloading, only a piece of the task is offloaded and the rest is computed locally. In [19], the authors implement partial offloading of the work, in which the IoT device computes the local component, and the offloaded component is performed in a virtual machine established within a mobile edge computing server. The authors of [20] employ horizontal offloading to benefit from the free computation resources of neighboring fog nodes. The task is divided into two sub-tasks, where one sub-task is locally computed and the other is computed at the helper fog node.

### 2.2. Partial Offloading to Multiple Helpers

Assume that a task can be divided into multiple independent sub-tasks, some of which are processed locally, and the remaining are offloaded to multiple fog nodes for parallel processing; then, the task execution latency will be reduced by many times. Take the case of face detection as an example, where an image can be partitioned into multiple subsets, and through parallel execution of these subsets with multiple devices, the speed of the detection procedure can be enhanced multiple times. The authors of [21] consider partial offloading in a meshed edge network using a heuristic technique based on graph theory. Tasks are partitioned based on projected delay in task queues and wait time in channel access. Work in [22] employs POMH to relieve workload from the Data Center (DC) in a busy road situation with slow-moving cars. Using the Lagrange method, the DC discovers free computation resources in all cars within its coverage zone and offloads proportionally sized task to them while taking their distance into account.

The author of [10] consider Vehicle-to-Vehicle (V2V) communication, in which a vehicle does parallel offloading of the task to other cars in its immediate vicinity. The work uses Markov Decision Process (MDP) to choose the cars and determine the size of the offloaded task for each vehicle. The research in [23] considers a 5G scenario in which a mobile device is served by a macro base station under a small base station. The task is parallel processed at the macro base station and a small base station. The size of sub-tasks is determined, considering task queue length at the base station and communication link quality of the mobile device, using Deep Reinforcement Learning (DRL) with MDP. The work in [24] used an iterative heuristic algorithm to make the offloading decision and to divide every task into three sub-tasks to be processed locally, at the edge server, and in the cloud server, respectively.

Work in [25] considered both horizontal and vertical offloading of the task in all layers except the cloud server. Task distribution is considered as a tree with branches to regulate the direction of the flow of tasks. For each node in the tree, the branch and bound algorithm are used to convert MINLP into NLP sub-problems. Each sub-problem is iteratively selected and solved using a depth-first search strategy. The work in [26] used directed acyclic task graphs to perform horizontal offloading of workloads among fog nodes. For each fog node, a clout value is calculated based on the fog node reliability (i.e., experience, residual power, computation capability, storage capacity, wait time, and distance). The task is offloaded to the fog node with the highest clout value. The authors of [27] subdivided tasks into multiple sub-tasks and formulated a Generalized Nesh Equilibrium Problem (GNEP) to solve the optimization problem. In this work, fog nodes advertise their tasks, for which helping fog nodes offer their resources. Based on offered resources, the advertising fog node calculates optimal solutions and does parallel offloading of the task.

The author of [28] discussed vertical task offloading to a cloud server as well as horizontal task offloading from task fog nodes to helper fog nodes. Task fog nodes advertise their tasks, which helps fog nodes offer their resources while considering channel rate. The authors used a many-to-one matching technique to match task node tasks with helper fog nodes. The task fog node sets offloading ratios for local task computing, cloud computation, and helper fog node computation. The authors of [12] performed POMH task offloading by leveraging both horizontal task offloading to neighboring fog nodes and vertical offloading to the cloud. They suggested a broad framework to minimize delay in service provisioning through an adaptive task offloading mechanism.

The authors of [29] discussed the impact of the number of task splits on time efficiency in POMH. The authors of [30] developed offloading policies based on residual energy with the fog nodes to optimize time or energy by offloading tasks to high-residual-power fog nodes and fog access points using POMH.

### 2.3. Externalities Problem

Solving the externalities problem in matching algorithms has been a significant research area for a long time, and scholars have contributed useful work in this area. In matching theory, the externalities problem describes a scenario in which one or both player sets have dynamic preference profiles that regularly change during the matching process. The matching decision of one player affects the preference order of every other player in the network. Stability in matching assignments is a prerequisite for using matching theory to solve resource allocation problems. Stability in matching assignment implies that the predetermined objective functions have been achieved, and matched partners are satisfied with their current partner and would not choose to switch partners. With externalities problems, it gets challenging to achieve stability in matching decisions.

Researchers working to solve the externalities problem propose that it is not essential that small changes in the preference profile of a player affect the stability of all matched pairs. This means that it is highly probable that only a few matching decisions result in players opting to change their partners. Based on this, researchers provide different ways for updating matches to satisfy all blocking pairs and maintaining stability to address the externalities problem. While updating matches by satisfying blocking pairs, the most difficult challenge is to control the domino effect. This reflects a situation in which satisfying one blocking pair may spawn more blocking pairs, changing this process into a cyclic process in which pairs begin to replicate themselves.

Knuth [16] believes that updating a stable matching using the Gale–Shapley algorithm is impossible due to the cyclic nature of the externalities problem. In contrast, Roth and Vande [31] established in their work that stable matching is always attainable when tackling externalities problems, even if we start from an arbitrary value. In contrast to the Gale–Shapley algorithm, which consistently provides the same stable matching outcomes, the Roth and Vande method offers a diversity of stable matching in each iteration, i.e., every time they give a different stable matching result. Jinpeng Ma [17] further deliberated the work of Roth and Vande and developed a stable matching update mechanism for the Gale–Shapely algorithm. The JM algorithm evaluates each stable match in turn and resolves its blocking pairs under the externalities problem. He demonstrated that the JM algorithm can always find stable matching with a probability of one. The work in [15] further deliberated the JM algorithm to reduce the stable match update time.

## 3. System Model

**Figure 1:**
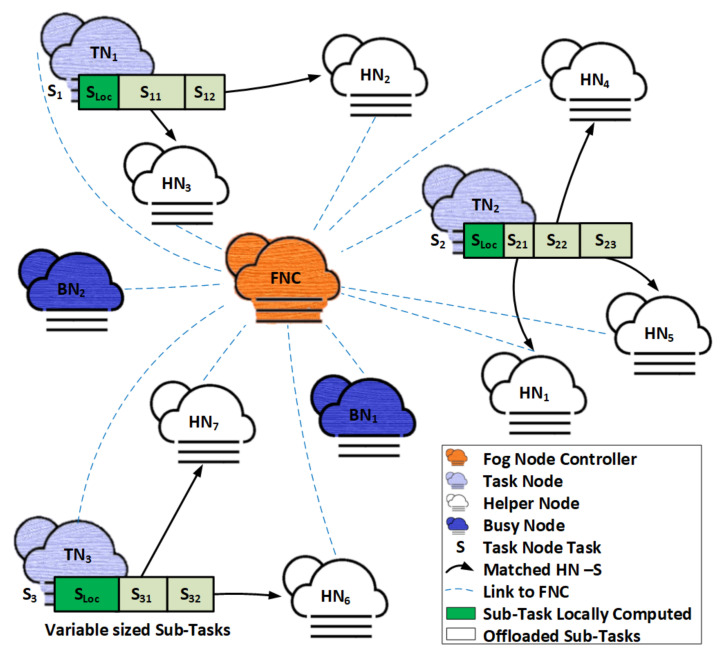
System model.

Consider a cooperative and self-sufficient fog-computing network in which fog nodes assist each other in accomplishing computing tasks. We assume that all fog nodes in this network have comparable computational capacity but differ in workload. Because of the difference in workload, the role of fog nodes can be altered from (1) Task Nodes (TNs) when they have a large task to compute, to (2) Helper Nodes (HNs) when they have some spare computation resources to compute tasks for others, and to (3) Busy Nodes (BNs) when they are busy computing previous tasks. The fog network in Figure 1 has three TN tasks, seven HNs, and two BNs.

Let there be *k* number of HNs and *m* number of TNs, denoted as $K=\left\{1,2,...,k\right\}$ and $M=\left\{1,2,...,m\right\}$, respectively. Let $W_{m}$ be the task size generated by TN, which we assume to be generically split-able, e.g., tasks such as face identification and image processing that may be divided into any number and size of sub-tasks [27]. Let $S_{m}$ represent the sub-tasks of $W_{m}$. For POMH task execution, $W_{m}$ is broken into r+1 sub-tasks. One sub-task is locally processed at the TN, represented by task percentage $\alpha_{loc}$, whereas the remaining *r* sub-tasks, represented by task percentages $\alpha_{k}$, are offloaded to *r* number of HNs for parallel computation of the task. For task percentages, the following condition must be met:

(1) $$\alpha_{loc}+\sum_{k=1}^{r}\alpha_{k}=1$$

In this study, we contemplate a centralized mechanism for task and resource allocation, for which a fog node is designated as the Fog Node Controller (FNC). TN, on generation of task $W_{m}$, sends an offloading request to FNC in the form of a tuple $\left(W_{m},C_{r},T_{m}^{FL}\right)$, where $W_{m}$ (in bits) represents input task size, $C_{r}$ (cycles) is the number of Central Processor Unit (CPU) cycles required to complete the task and, $T_{m}^{FL}$ (seconds) is the time required by TN to locally compute the entire task $W_{m}$. We assume that TN has sufficient resources to complete the task within task deadline $T_{m}^{max}$ and is employing POMH to improve task completion time, i.e., TN wants to improve $T_{m}^{FL}$ of task $W_{m}$.

We assume that FNC has Channel State Information (CSI) and resource information of all fog nodes in the network. FNC uses a many-to-one matching technique for resource allocation and task distribution in the network. TN tasks are split into multiple sub-tasks, whereas available free computation resources of an HN are considered as a single unit and allocated in full. When doing POMH, we need to ensure that all sub-tasks of task $W_{m}$ finish at the same time. For this, we need to size the sub-tasks such that TN and all HNs computing these sub-tasks complete them simultaneously.

### 3.1. Latency Model

In POMH, a task $W_{m}$ is simultaneously processed at TN and multiple HNs. As a result, the task completion time of $W_{m}$ is determined by several interconnected parameters and can be generally defined as local computation time and offload computation time. Following are the sequential steps involved in this process:

#### 3.1.1. Local Computation Time

Local computation time refers to the amount of time spent by TN in computing the local component of task $W_{m}$. In this paper, we assume that a TN can compute the local component of the task while offloading other sub-tasks to respective HNs. If $C_{m}$ is the Central Processor Unit (CPU) speed of TN${}_{m}$, and $\alpha_{loc}$ is the task percentage of task $W_{m}$ locally processed, then the latency incurred in computing the local component of the task can be calculated as:

(2) $$T_{m}^{l}=\frac{\alpha_{loc}W_{m}C_{r}}{C_{m}}$$

#### 3.1.2. Offload Computation Time

Steps involved in computing sub-tasks of task $W_{m}$ at multiple HNs are explained below:

1. Sub-task transmission time  
  This refers to the amount of time spent in transmitting sub-tasks of task $W_{m}$ from TN to HNs. Each sub-task will experience a different transmission time, dictated by the transmission rate $R_{k}$ between TN${}_{m}$ and HN${}_{k}$. Sub-task transmission time and transmission rate can be calculated as:
  (3) $$T_{k}^{t}=\frac{\alpha_{k}W_{m}}{R_{k}}$$
  (4) $$R_{k}=B_{k}log_{2}\left(1+\frac{P_{m}^{t}g_{k}}{\sigma^{2}}\right)$$
  where $P_{m}^{t}$ is the transmit power of TN${}_{m}$, σ is the white-noise power, and $g_{k}$ is the channel gain, which is inversely proportional to the distance between TN${}_{m}$ and HN${}_{k}$ [32].
2. Sequence time  
  In this paper, we assume that each fog node has a single antenna that can only transmit to one other fog node at a time. Therefore, in POMH, TN must send *r* distinct transmissions in *r* directions to offload *r* number of sub-tasks. This means that all sub-tasks will be communicated serially, and each sub-task will have to wait in TN for its turn to be offloaded, which we refer to as sequence time. The sequence time of a sub-task is defined as the sum of the transmission time of all sub-tasks offloaded before it. The sequence time for the first sub-task to be transmitted is zero, and the sequence time for subsequent sub-tasks can be calculated as follows:
  (5) $$T_{seq}=W_{m}\sum_{k=1}^{k-1}\frac{\alpha_{k}}{R_{k}}$$
  In this paper, we assume that HNs can start processing the sub-task from the moment they receive the sub-task. If *k* is the last sub-task of task $W_{m}$, then total transmission time of all sub-tasks of $W_{m}$ as calculated from Equations (3) and (5) is:
  (6) $$T_{m}^{tx}=W_{m}\sum_{k=1}^{r-1}\frac{\alpha_{k}}{R_{k}}+\frac{\alpha_{k}W_{m}}{R_{k}}$$
    
3. Queuing delay at HNs  
  This refers to the amount of time a sub-task must wait at HN while HN is busy computing other tasks. In this paper, only free computation resources of an HN are made available to compute for a single TN only. As a result, there will be no queue time in HN, and the sub-task will be computed as soon as HN receives it.
    
4. HN computation latency  
  This refers to the amount of time spent computing the sub-task at the fog node. If $C_{k}$ is the CPU cycles made available by HN${}_{n}$ for $\alpha_{k}W_{m}$ sub-task, then:
  (7) $$T_{k}^{c}=\frac{\alpha_{k}W_{m}C_{r}}{C_{k}}$$
    
5. Result download latency  
  This refers to the time spent transmitting the processed output from the HN to the relevant TN. In this paper, we assume that the output is very small compared to the input; therefore, download latency is neglected [33].
    
6. Total offload computation delay  
  The total time spent computing a sub-task at HN is the sum of the times mentioned above and can be given as:
  (8) $$T_{k}^{m}=\frac{\alpha_{k}W_{m}}{R_{k}}+\frac{\alpha_{k}W_{m}C_{r}}{C_{k}}+W_{m}\sum_{k=1}^{k-1}\frac{\alpha_{k}}{R_{k}}$$

#### 3.1.3. Total Task Latency

Because each sub-task of task $W_{m}$ has a different size, communicates over a variety of communication channels, and is processed at various fog nodes with diverse computational capabilities, each sub-task will have a variable latency. From these variable finish times, the sub-task taking the longest time to complete will define the latency of task $W_{m}$ as:

(9) $$T_{m}=max\left\{T_{m}^{l},T_{k}^{m}\right\}$$

For successful completion of task $W_{m}$:

(10) $$T_{m}^{}\leq T_{m}^{max}$$

### 3.2. Same Finish Time for All Sub-Tasks

Typically, fog networks have limited computation resources, so it is critical to make optimal use of this valuable resource. From Equation (9), we know that task $W_{m}$ completion time is decided by the sub-task that takes the longest time to complete. Therefore, if all sub-tasks of $W_{m}$ do not complete at the same time, the valuable computation resources of HNs that complete their share of $W_{m}$ earlier would be squandered. Therefore, for effective utilization of HN resources, all sub-tasks of $W_{m}$ must finish at the same time, i.e.,

(11) $$T_{m}^{l}=T_{1}^{m}=T_{2}^{m}=...=T_{r}^{m}$$

Except for the sub-task sizes, all criteria determining sub-task completion time are fixed. Therefore, we must regulate the sub-task sizes to ensure that all sub-tasks of $W_{m}$ are complete at the same time. This means that in POMH, all α’s of task $W_{m}$ are interdependent, and their relative sizes must be adjusted taking into account channel conditions and computation capabilities of devices on which they will be computed to ensure simultaneous completion of all sub-tasks. For a single task split, the values of $\alpha_{loc}$ and $\alpha_{k}$ for same completion time are determined by equating Equations (1), (2) and (8):

(12) $$\alpha_{k}=\frac{\alpha_{loc}R_{k}C_{r}C_{k}}{C_{m}\left(C_{k}+R_{k}C_{r}\right)}$$

(13) $$\alpha_{loc}=1/\left(1+\frac{R_{k}C_{r}C_{k}}{C_{m}\left(C_{k}+R_{k}C_{r}\right)}\right)$$

### 3.3. Problem Formulation

The objective of this paper is to make the best use of HNs’ available free computation resources to reduce TN task completion time and hence improve user experience. For this, we want to formulate a task-splitting and resource-allocation strategy based on a matching technique that efficiently matches sub-tasks of task $W_{m}$ to HNs, hence minimizing task computational latency. The optimization problem can be stated as follows:

   Problem (P1):

$$\begin{array}{cc} \; & \;\min_{}\;T_{m} \end{array}$$

(14) $$\begin{array}{cc} \; & \;s.t.\;T_{m}\leq T_{m}^{max} \end{array}$$

(15) $$\begin{array}{cc} \; & \;T_{m}<T_{m}^{FL} \end{array}$$

(16) $$\begin{array}{cc} \; & \;T_{m}^{l}=T_{1}^{m}=T_{2}^{m}=...=T_{r}^{m} \end{array}$$

(17) $$\begin{array}{cc} \; & \;\alpha_{loc}+\sum_{k=1}^{r}\alpha_{k}=1\mathrm{and}\forall \alpha \in \left\{0,1\right\} \end{array}$$

(18) $$\begin{array}{cc} \; & \;|S_{m}|=|HN\in W_{m}|+1 \end{array}$$

(19) $$\begin{array}{cc} \; & \;|S_{m}\in {HN}_{k}|\leq 1 \end{array}$$

Constraint (14) ensures that task $W_{m}$ is completed before its task deadline $T_{m}^{max}$, whereas constraint (15) ensures that overall task completion time is less than the time required to fully compute the task at TN. Constraint (16) ensures that all sub-tasks are complete at the same time. Constraint (17) ensures that whole task $W_{m}$ is converted into sub-tasks, i.e., no part of $W_{m}$ is left unattended. Constraint (18) ensures that no additional sub-tasks are created. The number of sub-tasks is equal to the number of HNs that will compute task $W_{m}$ with the TN, plus one that will be computed locally by the TN. If no HN commits to computing $W_{m}$ with TN, the task will be computed entirely by TN.

Constraint (19) ensures that only one sub-task of TN${}_{m}$ is offloaded to HN${}_{k}$.

The formulated problem represents a combinatorial optimization problem, which is NP-hard to solve [34]. It is nearly impossible to achieve an optimal solution in polynomial time for an increasing number of TNs and HNs. Moreover, each device aims to maximize its benefit, which may lead to an unstable outcome.

## 4. Proposed Solution

In this section, we explain the proposed technique to solve the problem formulated in Section 3.3. Due to the interdependence of multiple parameters, the formulated problem is NP-hard, and it is difficult to find its solution in polynomial time. Using traditional optimization-based techniques to solve such a problem is likely to result in extensive computation delays and may not work efficiently with a large number of devices. Matching theory, on the other hand, is scalable, computationally inexpensive, and simple to implement, and it has gained momentum in a variety of resource allocation problems [13]. It is an important mathematical tool for dynamically modeling and solving task offloading problems. It considers resource-demanding and resource-allocating devices to be members of two independent sets and builds beneficial associations between them while considering individual preference ranking over the players of the opposite set. As a result, each agent is satisfied and has no incentive to change its assigned allocation.

From Equation (11), we know that for efficient utilization of assigned HN computation resources, all sub-tasks of task $W_{m}$ must complete at the same time. To do this, the size of sub-tasks needs to be adjusted proportionately to the computation resources allocated to $W_{m}$. This indicates that when we use the matching theory for resource allocation in POMH, the size of sub-tasks will vary with every allocation and cancellation of a potential match. This change in sub-task size changes the HNs’ time and energy estimates for TN tasks, generating variations in HN preference order. In matching theory, this is an example of externalities, in which HNs have dynamic preference profiles, and the decision of one HN influences the decision of all HNs.

The externalities problem in matching theory necessitates special attention and the use of specialized algorithms to minimize the effects of constantly varying preference ordering of players. In this research, we employ the Deferred Acceptance Algorithm (DAA) to obtain initial stable matching assignments while ignoring the externalities problem. This stable matching assignment is then updated to solve the externalities problem using the proposed SMUA, which uses the stable-matching-update technique of the JM algorithm. The JM algorithm is designed to solve the externalities problem for Gale Shapley, a one-to-one matching technique that produces stable matching assignments in polynomial time with an algorithm convergence probability of one. We used the same technique to solve the many-to-one externalities problem for resource allocation in POMH. The proposed SMUA is a many-to-one resource allocation technique for POMH that always gives stable matching assignments.

### 4.1. Matching Game

In general, a matching game is a two-sided assignment problem between two disjointed sets of players, with each player having a defined preference order against players from the opposite set. The preference order specifies the extent to which a player’s objective functions are met by players from the opposing set. In our case, we have two sets of HNs and TN tasks represented by H and S, respectively, and we want to match TN tasks to available free computation resources of HNs so that the objective functions of both HNs and TN tasks are met. Before explicitly explaining the proposed solution, we explain the matching concepts in light of the formulated problem:

#### 4.1.1. Matching Assignment

A many-to-one matching assignment between H and S is based on a mapping function λ such that:

(20) $$\lambda \left(S_{m}\right)\subseteq H\mathrm{and}\;|\lambda \left(S_{m}\right)|\leq r_{m}^{}$$

(21) $$|\lambda \left(S_{m}\right)\subseteq H_{k}|\leq 1$$

(22) $$\lambda \left(H_{k}\right)\subseteq S\;\mathrm{and}|\lambda \left(H_{k}\right)|\leq 1$$

(23) $$H_{k}\in \lambda \left(S_{m}\right)\Leftrightarrow S_{m}\in \lambda \left(H_{k}\right)$$

Condition (20) and (21) show that a task can have a maximum of $r_{m}^{}$ matches with $r_{m}^{}$ number of HNs, while a task cannot have more than one match with a single HN. Condition (22) shows that an HN can only have one match, whereas, condition (23) implies that a task is matched to an HN if and only if that HN is matched to that task and vice versa.

#### 4.1.2. Association between HN and TN Tasks

*“In matching theory, an association set is defined for each player and is populated by those players from the opposite set with whom it may make an acceptable match, i.e., the pair meets defined objectives under specified constraints [35]."*

In our paper, if a pair of TN task and HN can jointly improve task completion time $T_{m}$ to make it lower than local computation time $T_{m}^{FL}$, then the pair can be associated. The association set is used to narrow the search space for matches, where a match for every player is searched from its association set only. Let $H_{k}^{A}$ and $S_{m}^{A}$ be association sets defined for HNs and TN tasks respectively. Then, HN can be defined in the association set of a TN task if and only if that TN task is also defined in the association set of that HN, i.e.,

(24) $$H_{k}\in S_{m}^{A}\Leftrightarrow S_{m}\in H_{k}^{A}$$

#### 4.1.3. Player Preference Profile

*“A matching game has two sets of preference relations ${≻}_{H}$ and ${≻}_{S}$ that allows each player ($H_{k}\in H)$ to indicate preference over all players ($S_{m}\in S)$ in the opposite set and vice versa [36].”*

The objective of this paper is to minimize task computation time $T_{m}$. Therefore, a TN task will prefer an HN with which it can jointly obtain the smallest task computation time $T_{m}$, i.e.,

(25) $$H_{k}{≻}_{S}H_{k^{\prime }}\Leftrightarrow T_{m}\left(H_{k}\right)<T_{m^{{}^{\prime }}}\left(H_{k^{{}^{\prime }}}\right)$$

The objective of HNs is to minimize $T_{m}$. In this paper, we also want to maximize the number of TN tasks leveraging HN computation resources. Therefore, rather than using the traditional profiling technique in which HNs aim to reduce their task computation time only, we use the metric of the percentage improvement in task completion time to define the HN preference profile. This technique calculates task completion time for two scenarios: (1) when the TN task is not served by HN and (2) when the TN task is served by HN. The difference in time is converted to a percentage improvement in task completion time. The TN task with the greatest percentage improvement in task completion time is preferred over the others.

(26) $$S_{m}{≻}_{H}S_{m^{\prime }}\Leftrightarrow \%T_{m}\left(S_{m}\right)>\%T_{m^{{}^{\prime }}}\left(S_{m^{{}^{\prime }}}\right)$$

The time calculations and corresponding preference order of TN tasks for HN change with each matching decision. These variations arise because the size of α of task $W_{m}$ changes with each addition and deletion of a match to finish all sub-tasks of $W_{m}$ at the same time, according to constraints (16) and (17) of the formulated problem. This introduces the externalities problem into the matching process, which will be discussed later in this section. TN tasks, on other hand, have a fixed preference profile.

#### 4.1.4. Quota/Capacity of Players

A player’s quota represents the number of matches that can be made with players of the opposite set. In this article, all free computation resources of an HN are allotted to a single TN task; hence, the HN quota is one. A TN task, on the other hand, is divided into r+1 sub-tasks and has a quota of *r* matches with HNs.

#### 4.1.5. Blocking Pair

*“A matching function λ is said to be blocked by a pair of agents $\left(H_{k},S_{m}\right)$ iff $H_{k}{≺}_{S_{m}}H_{k{}^{\prime }}$, $S_{m}{≺}_{H_{k}}S_{m^{{}^{\prime }}}$, but $S_{m}\notin \lambda \left(H_{k}\right)$, $S_{m^{{}^{\prime }}}\in \lambda \left(H_{k}\right)$ and similarly $H_{k}\notin \lambda \left(S_{m}\right)$, $H_{k^{{}^{\prime }}}\in \lambda \left(S_{m}\right)$, i.e., A pair $\left(H_{k},S_{m}\right)$ blocks assignment λ when they are not matched with each under current λ but they prefer to be matched with each other [37].”*

In other words, a player with a higher preference cannot be skipped to match with a player with a lower preference.

#### 4.1.6. Stable Matching Assignment

The stability of assignment λ implies that if $(S_{m},H_{k})\notin \lambda$ then atleast one of the two players $S_{m}$ and $H_{k}$ is better off in λ: either $S_{m}$ is matched with a player of H that $S_{m}$ prefers to $H_{k}$ or $H_{k}$ is matched with a player of S that $H_{k}$ prefers to $S_{m}$ [37].

Stability in matching assignments implies that all players are happy with their existing partners and would not want to change partners under the current circumstances. Stability can be achieved only when there is no blocking pair to the matched pairs.

### 4.2. Solving Externalities Problem for Resource Allocation in POMH Scenario

We use two algorithms to solve POMH-based resource assignment problems. The first algorithm finds stable matching assignments without addressing the externalities problem, while the second algorithm updates the first algorithm’s matching assignments to solve the externalities problem and obtain stable matching assignments.

#### 4.2.1. Stable Matching Assignments without Addressing the Externalities Problem

In our work, all algorithms are executed in FNC, and FNC makes resource allocation and task-split decisions based on the results of these algorithms. On generation of task $W_{m}$, TN sends an offloading request to FNC. FNC first establishes associations between HNs and TNs based on the transmission rate $R_{k}$ and the availability of adequate free computation resources with HN that can reduce $W_{m}$ computation time over its local computation time $T_{m}^{FL}$. FNC uses Channel State Information (CSI) periodically sent to it by all fog nodes to determine distances and transmission rates between the TN and HNs.

FNC then calculates preference profile $≻{}_{S_{m}}$ for all TN tasks. FNC also calculates the initial preference order of TN tasks for HNs $≻{}_{H_{k}}$ without taking the externalities problem into account. Based on this information, FNC uses DAA to match a single TN task to many HNs using the many-to-one matching technique. DAA generates stable matching assignments without addressing the externalities problem. The steps involved are shown in Algorithm 1 The externalities problem is then solved using the JM algorithm [17] to generate stable matching assignments in polynomial time with an algorithm convergence probability of one, as discussed below.
**Algorithm 1** Stable matching assignments without addressing the externalities problem
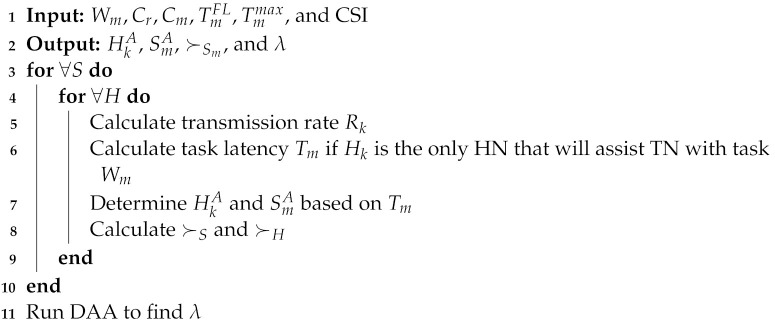


#### 4.2.2. Stable Matching Update Algorithm (SMUA) to Solve Externalities Problem

In this paper, the size of task $W_{m}$ sub-tasks changes with the allocation of HN resources. This is done to ensure that all sub-tasks of $W_{m}$ finish at the same time, which is essential for efficient utilization of assigned HN resources. HN preference order for TN tasks changes as sub-task sizes changes, resulting in a dynamic preference profile. This poses an externalities problem while making resource allocation decisions in POMH. If we try to solve the externalities problem during the matching process, the preference order of all HNs may begin to fluctuate, and we may never be able to find the stable solution as anticipated by Knuth [16].

**Figure 2:**
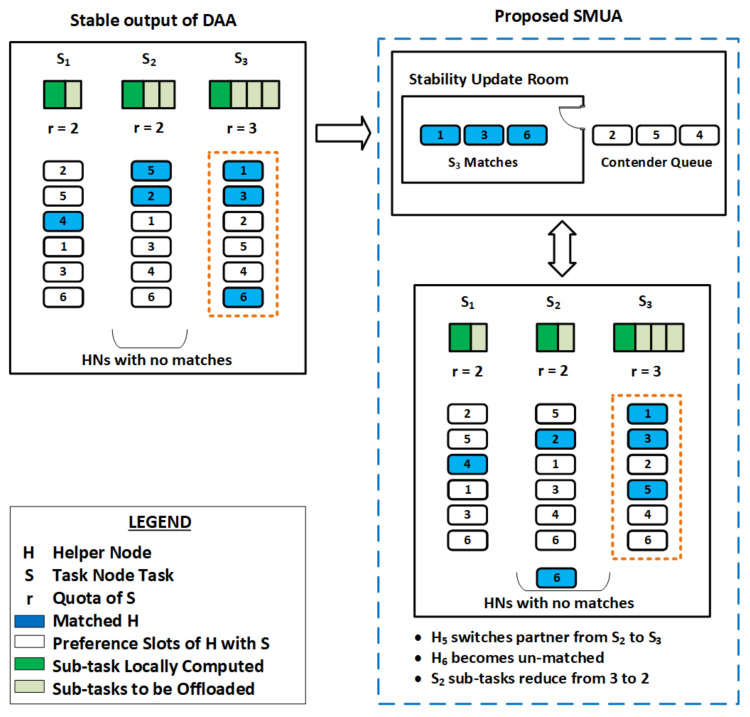
Proposed matching-update algorithm to solve externalities problem.

Therefore, we used the JM algorithm [17] to solve the externalities problem in this paper. The JM algorithm is a matching update process that is based on the concept that minor changes in a player’s preference profile may not affect the stability of all matched pairs. The JM algorithm starts with an arbitrary stable matching assignment produced by any matching technique and solves the externalities problem by resolving blocking pairs that may arise while the HN preference order changes. The JM algorithm always converges and finds stable matching with a probability of one. The JM method was designed to resolve externalities in one-to-one matching assignments; we modified the same approach to address externalities in many-to-one matching assignments. We attempt to update a stable matching by isolating the pairs responsible for its instability. This reduces the number of possible new blocking pairs as well as the size of the stable matching to update. The steps involved are shown in Algorithm 2 and, the working principle used in our proposed SMUA is given in Figure 2, and an explanation of the different steps involved is given below:

- Consider the three tasks $S_{1},S_{2}$, and $S_{3}$, each with a quota *r* of two, two, and three, respectively, as illustrated in Figure 2. The total allowable quota for TNs is seven, and there are six HNs to assist them. The left side of Figure 2 shows a stable match output of the DAA algorithm without addressing the externalities problem, with three tasks securing one, two, and three matches, respectively.
- SMUA solves the externalities problem by updating this non-externalities-based stable matching assignment with the JM technique. Matches to tasks are iteratively updated to account for new blocking pairs that may emerge as a result of the new HN preference profile until all externalities problems are solved and pairs become stable.
- Allow TN matches to enter a single-entry stability update room at random. In Figure 2, the matches of $S_{3}$, i.e., $H_{1},H_{3}$, and $H_{6}$, enter the stability update room.
- All HNs that can potentially form blocking pairs to matches of the selected TN task queue outside the stability update room in the sequence of ${≺}_{S_{m}}$. For $S_{3}$, HNs $H_{2},H_{5}$ and $H_{4}$ queue outside the stability update room.
- HN in front of the queue, i.e., $H_{2}$, enters the room and finds its preference number among the $S_{3}$ matches in ${≺}_{S_{3}}$. $H_{2}$ stays in the room for further calculations if its preference number is within quota *r* of $S_{3}$. Since $H_{2}$ is third in the preference order, $S_{3}$ matches against the quota of three; therefore, $H_{2}$ stays in the room.
- $H_{2}$ calculates the percentage time improvement if it serves $S_{3}$, and compares it to the percentage time improvement of its current match, i.e., $S_{2}$. If its current match has a high percentage time improvement, it continues with $S_{2}$ and exits the stability update room.
- $H_{5}$ enters the stability update room and finds that matching with $S_{3}$ provides a high percentage improvement in time over its current match $S_{2}$, so $H_{5}$ switches its match to $S_{3}$. The number of $S_{2}$ matches is decreased from two to one, and the number of sub-tasks is reduced from three to two.
- With the addition of the $H_{5}$ match, the total number of matches held with $S_{3}$ exceeds the permitted quota range of three. As a result, the match with the lowest preference in ${≺}_{S_{3}}$ is released, i.e., $H_{6}$ exits the stability update room. If a chance presents itself, $H_{6}$ will match another TN task.
- When $H_{4}$ enters the stability update room, it has a preference number of four amongst the held matches in ${≺}_{S_{3}}$. Since its sequence number is more than the $S_{3}$ quota, it quits the room.
- Iteratively, the process continues until the externalities problem is addressed and all blocking pairs are satisfied. When the matching assignment at the start of the iteration is the same as the matching assignment at the end of the loop, the finish condition is identified.

**Algorithm 2** Proposed SMUA to solve externalities problem

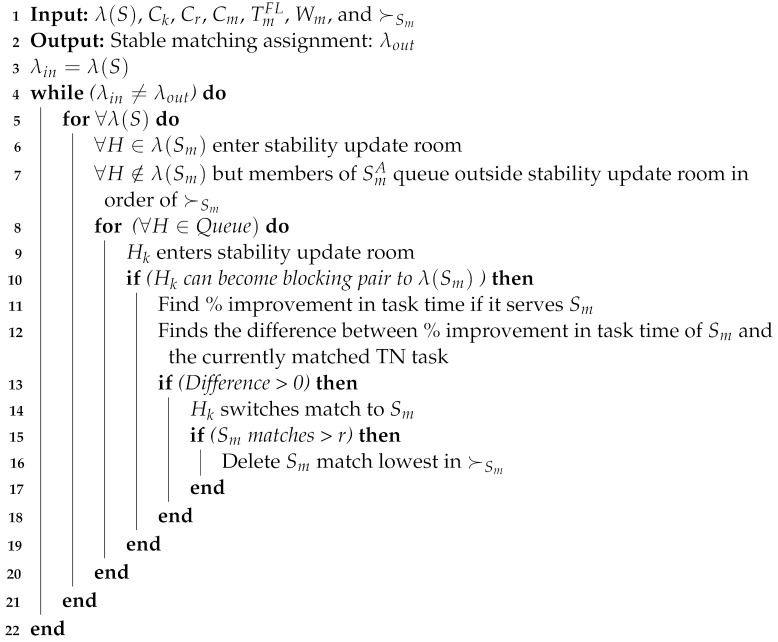



Since the proposed SMUA updates an existing stable matching assignment to solve the externalities problem by resolving new blocking pairs, it always produces stable matching assignments.

### 4.3. Complexity Analysis

The overall time complexity of our proposed SMUA depends on the following actions: (1) FNC works out the transmission rate between HNs and TNs, consuming a maximum of O(m×n) time; (2) FNC works out association sets and a preference profile of HNs and TN tasks for which it again requires O(m×n) time; (3) stable matching assignment without solving the externalities problem can be obtained in O(m×n) time; and (3) proposed SMUA will consume a maximum of O(m×n) time to update the matches to solve the externalities problem. Therefore the overall time complexity of the proposed SMUA can be expressed as O(m×n), which is polynomial.

## 5. Performance Evaluation

In this section, we evaluate the performance of the proposed SMUA by comparing our simulation results against other algorithms in the literature.

### 5.1. Simulation Setup

To demonstrate the efficacy of the proposed SMUA, we developed a MATLAB simulation of a cooperative fog network, and the values of significant parameters used in simulations are summarized in Table 2. The fog nodes are uniformly distributed over an area of 60 m × 60 m.

The role of fog nodes in our fog network alternates between HNs, TNs, and BNs. However, for simulation purposes, we consider 30 HNs and 5 to 15 TNs, with each simulation varying by 1 TN. Fog node computation resources (cycles/s) are considered to be heterogeneous and are uniformly selected from the range of 0.8–1.2 GHz (Here we are modeling smartphones with ARM Cortex-A8 processors as fog nodes. These smartphones have a 1 GHz clock speed [27]). TNs are configured to use all of their computation resources to compute their tasks, whereas HNs are configured to make only 80% of their computation resources available to compute TN tasks, with the remainder being allocated for internal processes. TNs generate tasks with uniform distribution in the range of 4000–5000 KB. Each bit requires 2500–3000 CPU cycles (cycles/s) to compute. (The data size and computational demand of face recognition applications are about 5000 KB and 3000 cycle/bit, respectively [27]).

We are employing a many-to-one matching technique to allocate HN resources to TN tasks. Therefore, in this paper, a TN task can be divided into 1–6 sub-tasks based on the number of matches obtained by a TN task. The free computation resources of an HN are treated as a single entity and allocated to a single TN task. After completion of the matching process, the size of sub-tasks is adjusted to ensure that all sub-tasks are complete at the same time. The effective communication range of HNs to compute TN tasks is set as 30 m. Each TN has an active up-link with dedicated bandwidth of 5 MB with each HN, ensuring no wait time to access the channel. The TN transmission power is set as 100 mW. Considering the PCS-1900 GSM band, the free space path loss in dB between a TN and an HN is calculated as: $PL_{m,n}=38.02+20\log_{}d_{m,n}$ [37]. The channel gain $g_{n}$ is then calculated as: $g_{n}=10^{-PL_{m,n}/10}$. The communication channel is assumed to be noisy, with noise power $\sigma^{2}=10^{-10}$.

### 5.2. Baseline Algorithms

To gauge the performance of the proposed SMUA, we compare its results with the following baseline schemes:

1. Liu et al. [27] (referred to as POST);
2. Zu et al. [28] (referred to as SMETO);
3. Zhang et al. [20] (referred to as FEMTO);
4. Local computing in which TNs process the task on a local device (referred to as Local).

The performance of the proposed SMUA against a non-externality-based matching solution will be discussed separately to gain insight into the factors influencing the performance of the proposed SMUA. Among the baseline schemes, SMETO models its resource allocation strategy as a matching game, whereas POST and FEMTO employ an optimization technique for resource allocation. POST, SMETO, and FEMTO all use the POMH technique to reduce task computation time. The primary objective of POST is task efficiency, whereas the primary objective of SMETO and FEMTO is energy efficiency. The DAA matching algorithm is used to make resource allocation decisions in POST and FEMTO.

### 5.3. Task Latency

**Figure 3:**
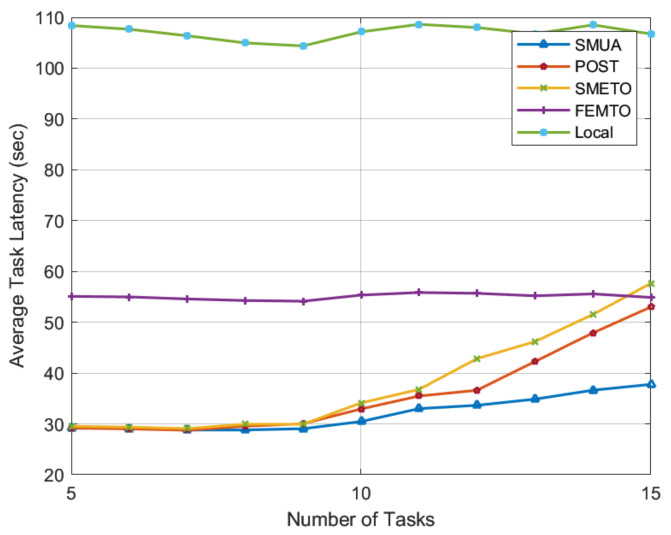
Average task latency with 3 matches permitted for each TN task.

Figure 3 shows the average task latency experienced by the baseline schemes under consideration. In this simulation, we permitted a maximum of three matches for each TN task. The number of TN tasks ranged from 5 to 15, while the number of HNs was set to 30. These are intriguing numbers for evaluating the performance of matching algorithms because when we test for TNs 5 through 9, the number of HNs exceeds the TN matching capacity, leaving many HNs unmatched. Every baseline algorithm performs optimally, selecting the best HNs to achieve their objectives. When there are 10 TNs, the number of HNs equals the TN task-matching capacity, and from TN 11 through 15, the number of HNs is fewer than the number that could serve the maximum capacity of the TN tasks. In such a case, baseline schemes become resource-constrained and exhibit their true performance in a heavy-workload scenario.

The results in Figure 3 show that the proposed SMUA outperforms all baseline schemes in both low- and high-workload scenarios. Such results can be attributed to two main reasons: (1) In this paper, rather than adopting traditional techniques for defining the HN preference profile, we used the metric of the percentage improvement in time to rank TN tasks. With this objective function, HNs prefer lonely tasks and seek to improve network time efficiency. The results show that our preference-profiling technique generates a matching trend that consistently outperforms all baseline schemes in both low- and high-workload scenarios, and (2) when we solve the externalities problem for POMH resource allocation, HNs become more agile in pursuit of their defined objective function. When solving the externalities problem, HNs make informed matching decisions based on the status of matches held with TNs; therefore, they can produce the most time-efficient results.

Among the baseline schemes, we observe that the objective of POST is time efficiency, whereas the objective of other schemes is energy efficiency. This disparity in HN objective functions impacts the time efficiency results in Figure 3. The results show that POST has a shorter task computation time than other baseline schemes. In low-workload scenarios, POST and SMRETO perform similar to SMUA until the number of HNs decreases in proportion to the maximum number of possible sub-tasks. FEMTO, on the other hand, seeks only one match to offload a portion of its task to a single HN, resulting in consistent performance in the current simulation settings.

### 5.4. Number of Non-Beneficial TNs

**Figure 4:**
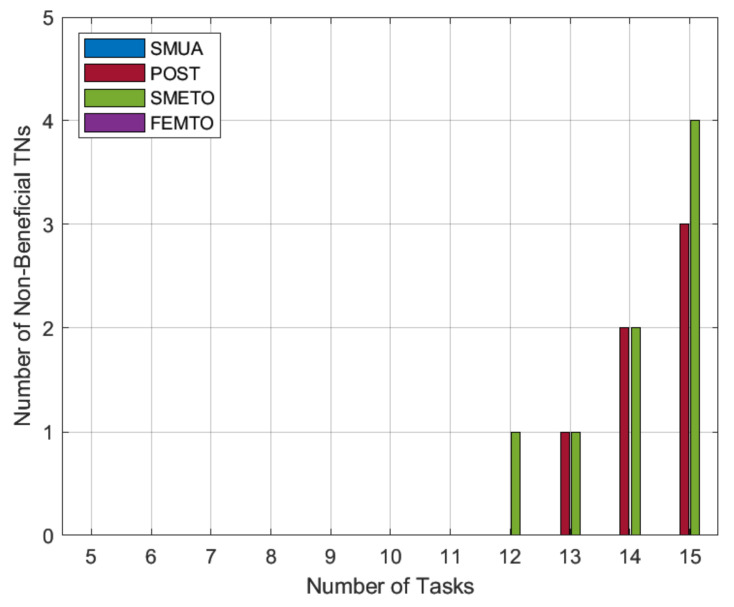
Non-beneficial TN tasks.

Figure 4 gives the number of TNs that did not benefit from the offloading process. This benefit is in terms of reduced task completion time as compared to local task computation time. This reflects the case where a TN task receives no matches and must compute the entire task $W_{m}$ locally. The results in Figure 4 reflect the dominance of our preference-profiling technique for HNs, in which a maximum number of TN tasks takes advantage of HN free computation resources. This happens because with our preference-profile technique, HNs favor lonely tasks, and hence serve the maximum number of TN tasks. On the other hand, the baseline schemes POST and SMRETO do not prioritize maximizing the number of beneficial TN tasks; the number of un-served TN tasks increases as the proportionate number of HNs decreases. The results also show that there is no non-beneficial TN task for FEMTO. This happens because FEMTO only offloads to a single HN, and when we map this requirement to present simulation settings, the number of available HNs is always more than the number of sub-tasks to be offloaded. As a result, FEMTO has no un-served TN task.

### 5.5. In-Depth Analysis of POMH Externalities

We aim to test the efficacy of our preference-profiling technique and test the factors that contribute positively and negatively to the effectiveness of solving externalities problems for resource allocation in POMH. As a result, we compare the performance of the proposed SMUA to (1) a non-externalities-based matching technique based on our preference-profiling technique for HNs (referred to as NEMA) and (2) a non-externalities-based matching technique based on the traditional preference-profiling technique for HNs (referred to as Gr-NEMA).

#### 5.5.1. Task Latency

Before delving into the results of the baseline schemes under review, let us first understand the types of preference profiles and their expected matching patterns that affect the simulation results. The traditional preference-profiling technique, Gr-NEMA, sets HN preference order in ascending order based on the amount of time required to complete the task. This preference-profiling technique is expected to generate a matching trend in which HNs prefer smaller tasks and tasks with more matches, potentially reducing the workload that HNs may need to perform. This means that HNs shy away from TN tasks, saving their own computation time while increasing network computation time. By contrast, we use the metric of the percentage improvement in time to rank TN tasks for HNs. With this preference profile, HNs prefer lonely tasks and seek to improve network time efficiency. When we solve the externalities problem with this preference trend, HNs become more agile in pursuit of their defined objective function and can produce the most time-efficient results.

**Figure 5:**
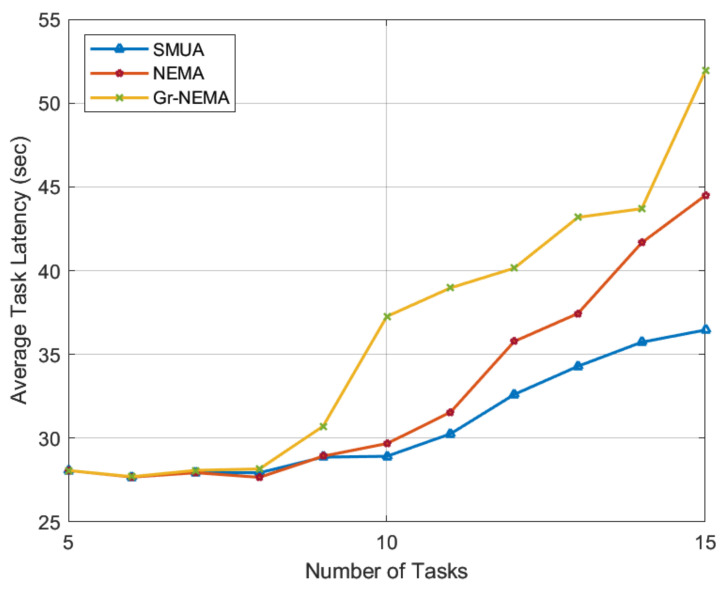
Average task latency with 3 matches permitted for each TN task.

The task latency results in Figure 5 are consistent with the corresponding matching trends, and we can observe that Gr-NEMA has the worst performance, since HNs were solely concerned with saving their own time and effort. We may reasonably deduce from the results that in Gr-NEMA, all HNs seek to lower their task completion time but end up working for longer periods. NEMA, on the other hand, has better task latency results due to a superior preference-profiling technique. We get the best time–task latency result when we solve the externalities problem and allow every HN to make an informed matching-update decision based on the matches held with TN tasks.

Though the proposed SMUA gives better results, it is important to note that from TN-5 to TN-9 in a low-workload scenario, there is a chance that non-externality-based matching techniques may give better time reduction. This is because the JM algorithm is derived from the Roth and Vande algorithm [31], and we know that the RV algorithm always produces different stable matching results depending on the sequence in which externalities are solved. Therefore, there is a chance that a sequence order may appear in a low-workload scenario when a non-externalities-based matching algorithm performs better than the proposed SMUA.

#### 5.5.2. Number of Non-Beneficial TNs

As previously explained, Gr-NEMA generates matching trends in which HNs prefer small tasks with more matches. In this configuration, the likelihood of a large number of non-beneficial TN tasks increases, whereas with the preference profile based on the metric of the percentage improvement in time, HNs prefer lonely tasks and seek to improve network time efficiency. Almost all TN tasks will be served in this configuration. If some are left un-served, it is the result of NEMA’s ill-informed decision-making. In contrast, SMUA always makes informed decisions and is expected to have few to no non-beneficial TN tasks.

**Figure 6:**
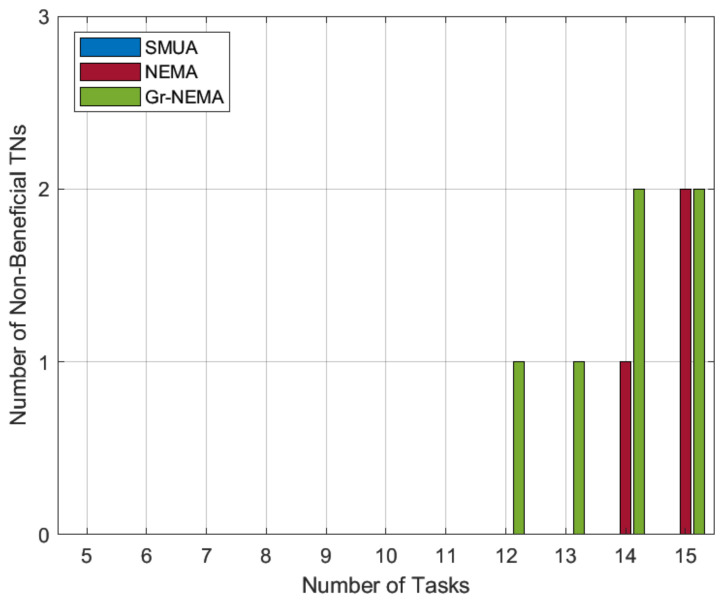
Non-beneficial TN tasks.

The results in Figure 6 confirm our predictions about the matching trends from the two preference-profiling techniques. The results suggest that preference profiling based on percentage improvement of time can also be used to reduce task outages. Reducing task outages refers to the situation in which we maximize the number of tasks served by HNs.

#### 5.5.3. Varying Quota of TN Tasks

**Figure 7:**
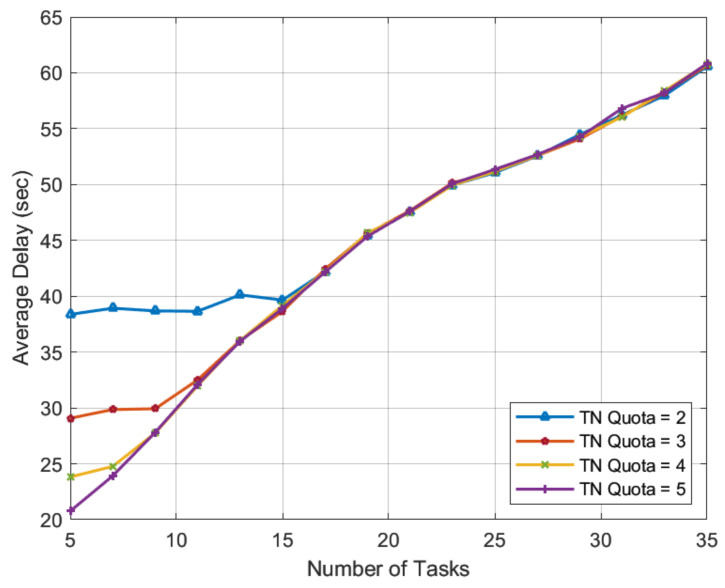
Average task latency with a varying quota of TN tasks.

The purpose of this simulation is to examine the effect of permissible TN task quota *r* on task latency and the number of non-beneficial TN tasks. For this purpose, we let the number of TN tasks range from 5 to 35 with 30 HNs and a TN quota *r* ranging from 2 to 5. These numbers depict a wide range of workload scenarios, from very low to very high. The results in Figure 7 show that with the HN preference-profiling technique proposed in the paper, the TN quota *r* has a significant impact on task latency only in low-workload scenarios. The greater the number of HNs that TN tasks can engage with its high quota, the shorter the task completion time. This advantage of the TN task quota is valid until the multiple of the TN task quota and TN tasks equals or exceeds the number of available HNs. This can be seen in Figure 7, where the impact of different TN task quotas *r* on task completion time nearly vanishes once the number of TN tasks reaches 15.

**Figure 8:**
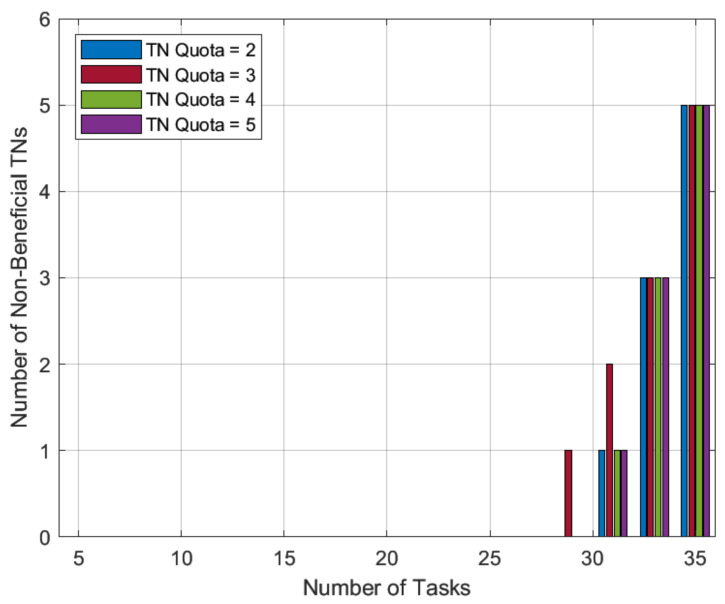
Non-beneficial TN tasks with varying quotas of TN tasks.

As we approach very-high-workload scenarios, i.e., for TN tasks ranging from 31 to 35, the number of HNs is insufficient to serve all TN tasks even with a single match. In this paper, we used a novel HN preference-profiling technique in the proposed SMUA, which is based on a percentage improvement in task completion time. This HN preference-profiling technique was expected to prefer lonely tasks and serve the greatest number of TN tasks. Except for TN tasks 29 and 31 with quota of 3, the results in Figure 8 are consistent with the expected matching trend, where we see that despite a wide range of allowable quotas *r* for TN tasks, HNs did not converge to match the same TN task. This deviation is a confirmation of RV findings of stable matching depending on the order in which externalities are solved [31]. Therefore, the occurrence of such an odd incident, especially near the border, is not surprising. We can safely conclude from the results that the proposed algorithm automatically adjusts to serve a maximum number of TN tasks while minimizing task completion time.

## 6. Conclusions

The paper proposes a new algorithm for parallel task offloading in IoT networks to improve task latency. The proposed technique uses many-to-one matching to solve the problem of mapping between sub-tasks at IoT nodes and computational resources at fog nodes. The proposed work further utilizes the JM algorithm to address externalities and to resolve blocking pairs due to dynamic preference profiles. Detailed performance evaluation is performed for the proposed technique and compared with different recently proposed techniques in the literature. Results highlight that the proposed technique improves task latency by 52% at high task loads. A further detailed evaluation of the proposed technique is presented to highlight the benefits of the algorithm’s key features, such as the preference profile technique, the use of the JM algorithm to resolve externalities, and the number of task-split selections.

## Figures and Tables

**Table 1:** Previous work on partial task offloading in fog computing and contributions of this paper.

Ref.	Objective	Solution Technique	PO	PO-MH	MT	Externalities
[19]	Maximize utility	Dinkel Bach method	✓			
[20]	Minimize energy + fairness	Heuristic algorithm	✓			
[21]	Minimize delay	Graph theory-based heuristic approach	✓	✓		
[22]	Minimize delay	Lagrange method	✓	✓		
[10]	Minimize delay	Markov Decision Process (MDP)	✓	✓		
[23]	Minimize delay + reduce sharing cost	Deep Reinforcement Learning (DRL)-based technique using MDP	✓	✓		
[24]	Minimize delay	Branch and bound algorithm-based heuristic approach	✓	✓		
[25]	Minimize delay + work balance	Graph theory is used with branch and bound algorithm	✓	✓		
[26]	Maximize utility	Directed acyclic task graphs are made for horizontal offloading	✓	✓		
[27]	Minimize delay + work balance	Fog nodes advertise the tasks, and helping fog nodes participate in bidding	✓	✓		
[28]	Minimize energy	Many-to-one matching theory. Fog nodes advertise the tasks, and helping fog nodes participate in bidding	✓	✓	✓	
[12]	Minimize delay	Adaptive task offloading mechanism	✓	✓		
[29]	Minimize delay	Adaptive task offloading mechanism	✓	✓		
[30]	Minimize delay or minimize energy	Formulates policies based on fog node energy to attain energy or delay minimization	✓	✓		
This work	Minimize delay	Many-to-one matching theory	✓	✓	✓	✓

Matching Theory (MT), Partial Offloading (PO), Partial Offloading–Multiple Helper (PO-MH).

**Table 2:** Simulation settings.

Parameter	Value
Number of HNs	30
Number of TNs	5–15
TN computation resources	U[0.8,1.2] GHz
HN computation resources	U[0.8,1.2] × 0.8 GHz
Input task size	U[4000, 5000] KB
Computational demand of a task	U[2500–3000] cycles
Dedicated bandwidth for up-link	$5\times 10^{6}$ Hz
TN transmission power	100 mW
White-noise power	$10\times 10^{-10}$ W

## Data Availability

Data is available from the corresponding author on request.

## Abbreviations

Notations used in the paper (explanations of sets with multiple values are provided with respect to the order in which the values are presented).

*H*, *S*	Set of HNs and TN tasks
*k*, *m*	Number of HNs and number of TNs
$W_{m}$, *r*	TN task size and number of sub-tasks to offload (quota)
$\alpha_{loc}$, $\alpha_{k}$	Percentage of task locally computed and offloaded to HNs
$T_{m}^{max}$, $T_{m}^{FL}$	Task deadline and task local computation time
$C_{k}$, $C_{m}$, $C_{r}$	HN CPU speed, TN CPU speed, and CPU cycles to compute one bit of task
$B_{k}$, $R_{k}$	Bandwidth between HN and TN and transmission rate
σ, $g_{k}$	White-noise power and channel gain
$P_{m}^{t}$	TN transmit power
$T_{k}^{t}$, $T_{seq}$	Sub-task transmission time and sequence time
$T_{m}^{tx}$, $T_{m}^{l}$	All sub-task transmission time and local computation time
$T_{k}^{c}$, $T_{k}^{m}$	HN computation time and HN latency for sub-task
$T_{m}$, *q*	Task total latency and quota of HN
${≻}_{H}$, ${≻}_{S}$	HN preference profile and TN task preference profile
$H_{k}^{A}$, $S_{m}^{A}$	Association set of $H_{n}$ with TN tasks and TN task $S_{m}$ with HNs
